# Impact of a Multichannel Blocker in Attenuating Intramyocardial Artery Remodeling in Hypertensive Rats through Increased Nitric Oxide Bioavailability

**DOI:** 10.1155/2019/6374582

**Published:** 2019-07-02

**Authors:** Begoña Quintana-Villamandos, María Jesús Delgado-Martos, Emilio Delgado-Baeza

**Affiliations:** ^1^Department of Anesthesiology, Reanimation and Intensive Care, Hospital General Universitario Gregorio Marañón, 28007 Madrid, Spain; ^2^Department of Pharmacology and Toxicology, Universidad Complutense de Madrid, 28040 Madrid, Spain; ^3^Department of Biomedicine, Universidad Francisco de Vitoria, 28223 Madrid, Spain; ^4^Molecular Biology Laboratory, Department of Experimental Medicine and Surgery, Health Research Institute of Hospital Gregorio Marañón, 28007 Madrid, Spain

## Abstract

Dronedarone is recommended for the treatment of atrial fibrillation. However, we do not know its effect on vascular remodeling. This study was designed to assess whether dronedarone has the potential to improve the intramyocardial artery remodeling induced by chronic hypertension. Ten-month-old male spontaneously hypertensive rats (SHR) were randomly assigned to receive dronedarone (100 mg/kg) or vehicle. Age-matched male Wistar-Kyoto rats served as controls. After 14 days of treatment, we studied the structure (geometry and fibrosis) of the intramyocardial artery using histological analysis. Nitric oxide (NO) in plasma was analyzed. In the untreated SHR, we observed a significant increase in external diameter, lumen diameter, wall width, cross-sectional area, and collagen volume density, as was expected in the experimental model. Dronedarone induced a significant decrease in wall width, cross-sectional area, and collagen volume density in SHR-D in comparison with untreated SHR. The values obtained in SHR-D were similar in the WKY control group. We found significantly higher NO levels in plasma in SHR-D than in untreated SHR. Dronedarone improves the intramyocardial artery remodeling induced by chronic hypertension in SHR through increased nitric oxide bioavailability.

## 1. Introduction

Hypertension leads to adverse remodeling (structural alterations) in the coronary artery wall and therefore increases the incidence of cardiovascular events [1, 2]. Antihypertensive therapy reverses vascular structural alterations and reduces cardiovascular morbidity [3, 4]. Therefore, the benefits by improving structural alterations in the coronary artery in patients with hypertension are an important factor in the selection of antihypertensive therapy [5].

Dronedarone is a novel antiarrhythmic drug for atrial fibrillation, acting as a multichannel blocker [6]. Dronedarone reduced the incidence of hospitalization due to cardiovascular events or death in patients with atrial fibrillation [7, 8]; however it is avoided in patients with unstable chronic heart failure [9]. In a preclinical study, we previously analyzed early regression of left ventricular hypertrophy following short-term use of antiarrhythmic agents and we found that dronedarone produces regression of left ventricular hypertrophy after two weeks of treatment [10]. However, its effect on intramyocardial artery remodeling has not been analyzed to date. Left ventricular hypertrophy, which is the usual complication of hypertension, is associated with structural changes, namely, coronary remodeling [11]; therefore we tested the hypothesis that short-term administration (two weeks) of dronedarone could improve the intramyocardial artery remodeling.

This study was designed to evaluate the efficacy of dronedarone in attenuating the intramyocardial branch of the obtuse marginal artery remodeling in spontaneously hypertensive rats (SHR), the most widely used animal models for human essential hypertension, left ventricular hypertrophy, and coronary damage.

## 2. Methods

### 2.1. Ethics

The study was performed in accordance with European Union Guidelines to use experimental animals (Directive 2010/63/EU and Spanish Law RD 53/2013) and was approved by the Ethics Committee of Hospital Gregorio Marañón, Madrid, Spain.

### 2.2. Animals and Experimental Protocols

Ten-month-old male SHR were randomly assigned to receive oral dronedarone (100 mg/kg once daily) (SHR-D, n=11) for a period of 14 days or to receive vehicle (SHR, n=11). A third group of normotensive control rats (WKY, n=11) was also added. Once the treatment was finished, the rats were killed by decapitation after sedation with diazepam 4 mg/kg and ketamine 10 mg/kg (intraperitoneal injection).

### 2.3. In Vivo Measurements (Arterial Pressure and Heart Rate)

As described in detail in previous studies [12] systolic arterial pressure (SAP) and heart rate (HR) were measured using the plethysmographic method on the tail artery of conscious animals (Niprem 546, Cibertec, Madrid, Spain). Several determinations were made before and after treatment, and the findings were considered valid if 10 consecutive measurements were within 10 mmHg of each other.

### 2.4. Intramyocardial Artery Structure

We analyzed 6 animals for each group as described in detail previously [12–14]. Hearts were excised and fixed in 4% paraformaldehyde. An equatorial cross section of the left ventricle was embedded in paraffin. Sections (3 sections/rat) were cut (thickness, 5 *μ*m) and stained with orcein [12]. The intramyocardial branch of the obtuse marginal artery (branch of the circumflex coronary artery) of the left ventricle was located, and the external diameter (ED) (lumen diameter + tunica intima + tunica media + tunica adventitia) and lumen diameter (LD) of the intramyocardial artery were measured as described [12]. Wall width (WW) was calculated as (ED−LD)/2, the wall-to-lumen ratio (W/L) as (WW/LD)×100, and the media cross-sectional area (CSA) (tunica intima + tunica media + tunica adventitia) as (*π*/4)×(ED2−LD2) [13]. For collagen staining, 5 *μ*m sections (3 sections/rat) of paraffin blocks were stained with picrosirius red, and the collagen volume density (CD) was determined using a stereological method [14].

### 2.5. Measurement of Plasma Nitrite Level

We analyzed 5 animals from each group. The same protocols described in previous studies [15] were followed in the present one. Plasma nitrite level was assessed using a Griess reaction-based protocol adapted from other authors [16, 17]. To eliminate thiols that interfere with the Griess reaction, 100 *μ*L of plasma or nitrite standard was mixed with 10 *μ*L of N-ethylenediamine. The protein from samples was precipitated by addition of 110 *μ*L of trichloroacetic acid. 50 *μ*L of supernatant (obtained by centrifugation) was mixed with 50 *μ*L of saturated vanadium chloride, 25 *μ*L of sulfanilamide, and 25 *μ*L of N-naphthyl-ethylenediamine. The mixture was incubated at 37°C for 1 hour, and absorbance was read at 540 nm.

### 2.6. Statistical Analysis

The results were expressed as median and 25th and 75th percentiles and analyzed with the Mann-Whitney* U* test (arterial structure parameters and nitrites). The results were expressed as mean ± SEM and analyzed using repeated-measures analysis of variance (physiological parameters). Statistical significance was set at* P* ≤ 0.05. The analysis was performed using IBM SPSS Statistics for Windows, version 20.0.

## 3. Results

### 3.1. Administration of Dronedarone Lowers Blood Pressure and Heart Rate

Rat weight was significantly higher in WKY than in SHR and SHR-D (460.40±1.12 vs. 401.22±3.02 g,* P*<0.01, and 460.40±1.12 vs. 380.51±6.21 g,* P*<0.001, respectively); however, the difference was not statistically different between SHR and SHR-D.

Dronedarone significantly reduced SAP and HR in SHR-D with respect to control SHR. SAP remained unchanged in SHR-D and WKY (Table 1).

### 3.2. Administration of Dronedarone Improves Structural Intramyocardial Artery Remodeling

**Figure 1:**
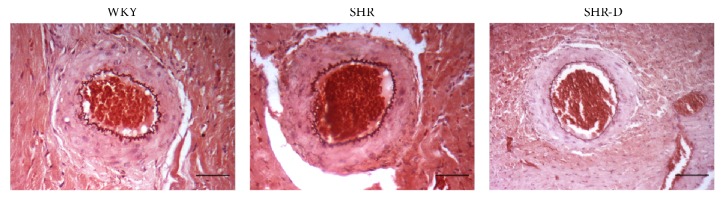
Histological sections of the intramyocardial branch of the obtuse marginal artery from WKY, SHR, and SHR-D (orcein, 100x, 100 *μ*m).

The effect of dronedarone on artery morphology is shown in Table 2 and Figure 1. SHR presented hypertrophic outward remodeling associated with a significant increase in CSA and LD in the intramyocardial branch of the obtuse marginal artery when compared with WKY. CSA and WW were significantly lower after 2 weeks of treatment with dronedarone in SHR-D than in SHR, and no differences were observed with respect to WKY. LD in SHR-D did not differ from that in SHR. ED was significantly increased in SHR when compared with WKY; however, administration of dronedarone significantly decreased this parameter in SHR-D when compared with SHR.

**Figure 2:**
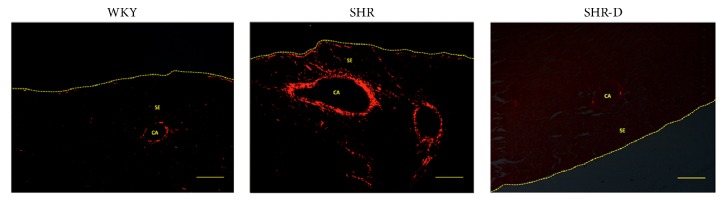
Images of collagen staining (picrosirius red, 40x, 200 *μ*m) of the intramyocardial branch of the obtuse marginal artery from WKY, SHR, and SHR-D. CA, coronary artery; SE, subepicardium.

The effect of dronedarone on intramyocardial artery fibrosis is shown in Table 2 and Figure 2. Vascular collagen was expressed as CD. Compared with WKY rats, the CD of the intramyocardial artery was significantly increased in SHR. Dronedarone significantly reduced the collagen fiber content, and the CD value was comparable to that of WKY.

### 3.3. Administration of Dronedarone Increases the Bioavailability of Nitric Oxide (NO)

Compared with WKY rats, the NO of the intramyocardial artery was significantly decreased in SHR. Dronedarone significantly increased the NO, and this parameter was comparable to that of WKY (Table 2).

## 4. Discussion

Our results show that two weeks of treatment with dronedarone improves the intramyocardial artery remodeling in adult SHR through increased nitric oxide bioavailability.

In the literature, we find that antihypertensive and antiarrhythmic drugs produce regression coronary artery remodeling after chronic treatment in SHR: administration of losartan for 5 weeks reduced wall thickness and CSA, amlodipine and enalapril led to a reduction in media thickness and CSA after 12 weeks, perindopril and indapamide led to a reduction in CSA and vessel diameter after 8 weeks, and lisinopril reduced the thickness of the coronary artery media after 12 weeks [18–21]. Furthermore, short-term administration of esmolol (48 hours) reduced wall thickness, CSA, and vessel diameter [15, 22]. However, the effect of dronedarone on vascular remodeling has not been investigated to date.

Functional and structural alterations of the coronary circulation have been well documented in left ventricular hypertrophy [1]. In a preclinical study, we show that short-term treatment (two weeks) with dronedarone has the potential to reverse the left ventricular hypertrophy induced by arterial hypertension in the SHR model of compensated ventricular hypertrophy [10]. In this context, we found it of interest to assess the impact of dronedarone in attenuating intramyocardial artery remodeling. And it is plausible that dronedarone induces changes in intramyocardial remodeling.

Dronedarone is a new drug (class III antiarrhythmic) used for the maintenance of sinus rhythm and control of ventricular rate [23]. Dronedarone produces a multichannel blockade of the sodium, potassium, and calcium channels and exhibits antiadrenergic properties [6]. Dronedarone reduces heart rate and blood pressure, and it has a direct cardioprotective effect (reduction in infarct size in an animal model of ischemia/reperfusion). Therefore, dronedarone reduces the risk of acute coronary syndrome [24]. This cardioprotective effect could be related to the improvement of structural alterations in intramyocardial arteries observed in the present study.

In the present study, the mechanism underlying dronedarone improving the intramyocardial artery remodeling could be related in part to an increase in the bioavailability of NO. Coronary artery remodeling in the presence of hypertension implies structural changes [25], but also endothelial dysfunction [26, 27]. Dronedarone acts on the coronary artery producing vasodilatation by activating the nitric oxide synthase pathway [28]. NO inhibits smooth muscle cell (SMC) proliferation of the arterial wall [29], decreasing wall thickness. Therefore, NO is a key regulator of cardiovascular remodeling thanks to its antihypertrophic and direct antiproliferative effects [30]. Our group has demonstrated that another antihypertensive drug (esmolol) reverses structural alterations in the coronary arteries (decreased media thickness and SMC count) by increasing the bioavailability of NO in SHR [15, 22]. With the data available from the literature, the mechanisms discussed above remain speculative and future studies are required to gain insight into the mechanism underlying dronedarone-induced protective effects on intramyocardial arteries remodeling.

Our results provide preclinical data on the beneficial effects of dronedarone on intramyocardial artery remodeling, which is associated with an increased incidence of cardiovascular events [1, 2]. During the last decade, dronedarone has been studied extensively in a wide range of experimental and clinical settings. These studies have revelated that dronedarone is an interesting antiarrhythmic agent for the treatment of atrial fibrillation [31]. Dronedarone reduces cardiovascular morbidity and mortality in clinically stable patients with other risk factors for recurrent atrial fibrillation [23]. Atrial fibrillation is the most common arrhythmia in clinical practice with significant morbidity and mortality [6]. In fact, atrial fibrillation induces ischemic microcirculatory flow abnormalities in the ventricle contributing to the risk of acute coronary syndromes [32]. Therefore, dronedarone is now widely used to treat patients with atrial fibrillation. However, regression of structural alterations in the intramyocardial arteries in patients with atrial fibrillation could play a key role in the selection of antiarrhythmic therapy. Further studies are needed to clarify the underlying mechanisms of positive effect of dronedarone on vascular remodeling.

The present study is subject to limitations. This study was designed to evaluate the efficacy of dronedarone in attenuating the intramyocardial branch of the obtuse marginal artery remodeling in SHR. Our results show that dronedarone improves structural intramyocardial artery remodeling; however, vascular remodeling in the presence of hypertension implies not only alterations in the structure of vessels, but also endothelial dysfunction [26, 27]. Thus, a major goal of research could be to analyze the effect of dronedarone on coronary artery vasodilator function. On the other hand, the complicated mechanisms underlying vascular remodeling involve not only decreased NO bioavailability. In fact, activation of the pathways of vascular smooth muscle contraction, vascular oxidative stress, and inflammation are implicated [27]. Therefore, we need to explore new mechanisms related to the positive effect of dronedarone on vascular remodeling.

In conclusion, this study suggests that dronedarone improves the intramyocardial artery remodeling induced by chronic hypertension in SHR through increased nitric oxide bioavailability.

## Figures and Tables

**Table 1:** Systolic blood pressure and heart rate from WKY, SHR, and SHR-D.

Groups	Time treatment (days)	SBP (mmHg)	HR (bpm)
WKY (n=6)	0	130 ± 21	414 ± 20
	14	137 ± 23	401 ± 24
SHR (n=6)	0	174 ± 15^*∗∗*^	395 ± 21
	14	180 ± 11^*∗∗*^	410 ± 18
SHR-D (n=6)	0	176 ± 21^*∗∗*^	398 ± 12
	14	141 ± 12^##^	301 ± 17^*∗∗∗*, ###^

SBP, systolic blood pressure; HR, heart rate. Values are shown as mean ± SEM. Statistically significant differences are shown (^*∗∗*^*P* < 0.01 versus WKY;  ^*∗∗∗*^*P* < 0.001 versus WKY; ^##^*P* < 0.01 versus SHR; ^###^*P* < 0.001 versus SHR).

**Table 2:** Geometry and collagen in the intramyocardial artery from WKY, SHR, and SHR-D.

Structural parameters	WKY	SHR	SHR-D
ED (*μ*m)	151 (150-219)	349 (312-377)^*∗∗*^	250 (248-303)^*∗∗*,#^
LD (*μ*m)	68 (60-129)	201 (110-212)^*∗*^	176 (144-226)^*∗*^
WW (*μ*m)	43 (36-47)	82 (71-107)^*∗∗*^	49 (38-52)^##^
W/L (%)	60 (37-74)	46 (33-80)	27 (18-36)^*∗*,#^
CSA (*μ*m^2^)	15058 (14381-21195)	70227 (54016-82310)^*∗∗*^	31885 (25215-38623)^##^
CD (%)	13.83 (11.81-21.40)	41.66 (40.68-52.78)^*∗∗*^	15.38 (12.57-17.25)^##^
NO (*μ*M)	1.76 (1.68-1.84)	1.35 (1.32-1.40)^*∗*^	1.66 (1.46-1.80)^ #^

ED, external diameter; LD, lumen diameter; WW, wall width; W/L, wall/lumen ratio; CSA, cross-sectional area; CD, collagen volume density; NO, nitric oxide. Data are expressed as median (25th-75th percentiles). Statistically significant differences are shown ( ^*∗*^*P*<0.05 versus WKY;  ^*∗∗*^*P*<0.01 versus WKY; ^#^*P*<0.05 versus SHR; ^##^*P*<0.01 versus SHR). Geometry and collagen (n=6) and NO (n=5).

## Data Availability

The data used to support the findings of this study are available from the corresponding author upon request.
